# Renal function deterioration in adult patients with type-2 diabetes

**DOI:** 10.1186/s12882-020-01952-0

**Published:** 2020-07-29

**Authors:** Peter Bramlage, Stefanie Lanzinger, Eva Hess, Simon Fahrner, Christoph H. J. Heyer, Mathias Friebe, Ivo Buschmann, Thomas Danne, Reinhard W. Holl, Jochen Seufert

**Affiliations:** 1 Institute for Pharmacology and Preventive Medicine, Bahnhofstrasse 20, 49661 Cloppenburg, Germany; 2 Institut für Epidemiologie und medizinische Biometrie, ZIBMT, Universität Ulm, Ulm, Germany; 3 Deutsches Zentrum für Diabetesforschung e.V, München-Neuherberg, Germany; 4 Diabetologische Schwerpunktpraxis Dres, Hess, Worms, Germany; 5 Medizinische Klinik, SRH Klinik Sigmaringen, Pfullendorf, Germany; 6 Diabetespraxis Viersen, Viersen, Germany; 7 Evangelisches Krankenhaus, Oberhausen, Germany; 8 Department of Angiology, Medical University of Brandenburg, Brandenburg, Germany; 9 Kinderkrankenhaus auf der Bult, Diabeteszentrum für Kinder und Jugendliche, Hannover, Germany; 10 Universitätsklinikum Freiburg, Medizinische Fakultät, Freiburg, Germany

**Keywords:** Type 2 diabetes, Chronic kidney disease, eGFR slope, Hypertension, Dyslipidemia

## Abstract

**Background:**

To explore, in a large group of patients with type-2 diabetes (T2DM), renal function decline in terms of the slope of the estimated glomerular filtration rate (eGFR) over time, and to find out how classical risk factors, such as the presence of hypertension, dyslipidemia and microalbuminuria, affect the renal function.

**Methods:**

The analysis included 32,492 adult T2DM patients from the DIVE/DPV registries who had serial eGFR determinations and information on the presence of microalbuminuria, hypertension and dyslipidemia available.

**Results:**

Patients had a mean age of 66.3 years, 52.6% were male with a mean BMI of 31.7 kg/m^2^. The mean eGFR was 78.4 ± 21.4 mL/min/1.73m^2^. The results showed that the prevalence of renal function impairment understood as chronic kidney disease (CKD) is considerable (53.0%) in a population of patients with T2DM and has a high incidence rate of 6.6% within a year. Serial determinations of the eGFR are, however, infrequent (7.8% of all patients) and these patients are characterised by the presence of a high-risk profile for CKD, such as hypertension (88.1%) and dyslipidemia (66.1%). Over a three-year time period, 30.9% of the patients had an eGFR slope of -12 mL/min/1.73m^2^ or more; and more than a doubled proportion of patients with an eGFR < 30 mL/min/1.73 m^2^ (3.8% vs. 1.8%; *p* < 0.001). Hypertension and albuminuria contributed to renal function decline while dyslipidemia did not negatively affect the slope.

**Conclusion:**

CKD is highly prevalent in patients with T2DM. Serial surveillance of the glomerular filtration rate is, however, not established in clinical practice, which would be necessary as indicated by a doubling of patients with an eGFR < 30 mL/min/1.73 m^2^ within 3 years. Moreover, the use of renin-angiotensin blocking agents was low, pointing at considerable room for improvement. Taken together we conclude that a closer surveillance of patients with diabetes based on the presence of further risk factors is mandatory combined with a mandatory prescription of RAS blocking agents once microalbuminuria and / or renal function deterioration develops.

## Background

Diabetes is the leading risk factor for the development of renal impairment and end-stage renal disease [1]. Irrespective of a potential causal relationship, chronic kidney disease (CKD), defined as an eGFR < 60 mL/min/1.73 m^2^ OR an eGFR ≥60 mL/min/1.73 m^2^ together with albuminuria (≥30 mg/g), affects approximately 50% of the patients with type 2 diabetes mellitus (T2DM) [2, 3].

CKD is usually regarded as progressive and may eventually lead to end-stage renal disease (ESRD)/kidney failure. Although risk management strategies, including blood glucose control, have resulted in a decline of cardiovascular sequelae, the frequency of ESRD in patients with diabetes remains virtually unchanged [4, 5]. A deeper understanding of the disease history and progression along with the development of new treatment strategies is mandatory to cope with the burden of ESRD worldwide [6].

We aimed to explore, in a large group of patients with T2DM, determinants of renal function decline, through assessment of the estimated glomerular filtration rate (eGFR) slope over time. Furthermore, we wanted to find out how classical risk factors, such as the presence of hypertension, dyslipidemia and microalbuminuria, would affect the further course and outcomes of T2DM patients in terms of their renal function.

## Methods

### Study design and data sources

This analysis used combined data from the DPV and DIVE registries [7, 8]. Their design has been described previously. In short, the DPV initiative collects data on patients with diabetes mellitus from centers predominantly located in Germany [8, 9]. Data are collected every 6 months using specific DPV software and the anonymized data are sent to the University of Ulm for aggregation into the database. The DPV initiative, which was established in 1995, was approved by the ethics committee of the University of Ulm, and data collection was approved by local review boards.

The DIVE registry was established in 2011 [2, 7, 10]. Consecutive patients with diabetes mellitus, regardless of their disease stage, were enrolled from centers across Germany, and continue to be followed up. Data are entered into an online database, which also uses the DPV software. The protocol was approved by the ethics committee of the Medical School of Hannover, and all patients included in the DIVE registry provided written informed consent.

Patients were sampled in March 2019 and included in the current analysis if they had T2DM, were at least 18 years old, initially registered between 2000 and 2017, and had an eGFR value calculated according to the Chronic Kidney Disease Epidemiology Collaboration (CKD-EPI) [11]. At least five eGFR measurements over a period of 3 years had to be available per patient.

### Documentation

For each patient, where data on eGFR was available, we aggregated data per patient for the first year with eGFR measurement (baseline) up to 3 years follow-up. CKD was defined as eGFR < 60 mL/min/1.73 m^2^ OR an eGFR ≥60 mL/min/1.73m^2^ and albuminuria (≥30 mg/g) [12, 13]. Hypertension was defined as blood pressure (BP) levels above 140 mmHg systolic (SBP) or 90 mmHg diastolic (DBP) or the receipt of antihypertensive drugs. Dyslipidemia was defined as an LDL-C cholesterol of ≥100 mg/dL without further risk factors and ≥ 70 mg/dL in patients with cardiovascular disease (CVD) or CKD or the receipt of lipid lowering drug treatment [14].

### Statistics

Categorical variables are presented as percentages. Continuous variables are presented as medians with first and third quartiles (Q1, Q3). Unadjusted comparisons were conducted using a Chi-squared or Kruskal–Wallis test. A *p*-value < 0.05 was considered statistically significant. The false discovery rate method was used to correct *p*-values for multiple testing.

eGFR-Slopes over the 3 years follow-up were estimated using a mixed linear regression model with a random participant intercept. eGFR-slopes were categorized into greater than (>)12 (indicating improvement), 0 up to 12, 0 down to − 12 and smaller than (<) -12 (indicating worsening). We used multivariable logistic regression models to analyze the association between eGFR-slope categories and albuminuria, hypertension and dyslipidemia. Models were adjusted for age, sex, diabetes duration and BMI. As a sensitivity analysis models were additionally adjusted for ACE-inhibitor (ACEi) and angiotensin receptor blocker (ABR) use. We also conducted analyses stratified by comorbidity. Statistical analysis was performed using SAS version 9.4.

## Results

**Fig. 1:**
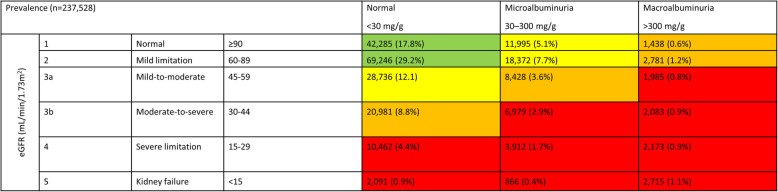
Chronic Kidney Disease prevalence by eGFR and albuminuria (based on [2, 12]). Green, low risk (if no other markers of kidney disease, no CKD); yellow, moderately increased risk; orange, high risk; red, very high risk

**Fig. 2:**
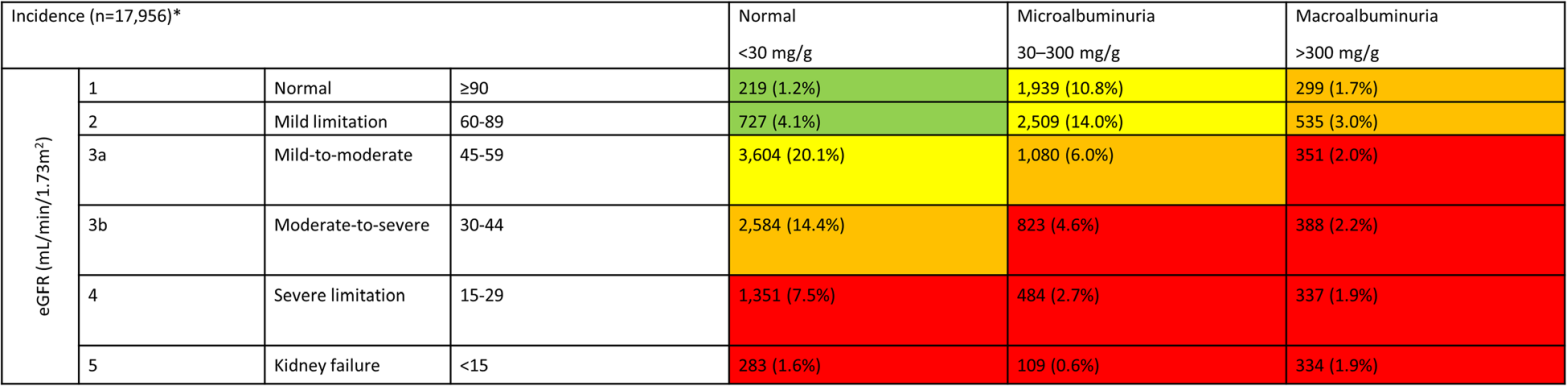
Chronic Kidney Disease incidence by eGFR and albuminuria (based on [2, 12]). Green, low risk (if no other markers of kidney disease, no CKD); yellow, moderately increased risk; orange, high risk; red, very high risk. *There were 27,201 patients with incident CKD during one year. For 9245 of these patients we were not able to group them into risk classes

The database included 413,239 adult patients with T2DM. For 237,538 patients, information on eGFR determinations and the level of albuminuria was available allowing the grouping of these patients into different CKD stages (Fig. 1). Of these patients, 91,411 had an eGFR of l < 60 mL/min/1.73m^2^, and 34,586 had an eGFR of ≥60 mL/min/1.73 m^2^ but with an albuminuria of at least 30 mg/g. As such, the prevalence of CKD was 53.0%. This value was slightly lower than in a prior analysis of the same dataset sampled one year earlier [2]. For 6.6% (27,201 of 413,239) of the patients, CKD was observed for the first time within in the last year of documentation (Fig. 2).

### Baseline characteristics

Of the 413,239 patients, 32,492 patients had at least five eGFR determinations within a time frame of three years and all variables available for the subsequent analyses. These patients had a mean age of 66.3 years and 52.6% were male with a mean BMI of 31.7 kg/m^2^ (Table 1). T2DM was diagnosed a mean of 11.3 years previously. The majority of patients were treated with metformin (56.7%), followed by insulin (45.8%), sulfonylurea (19.4%) and DPP-4 inhibitors (16.7%). Hypertension was documented for 88.1% of the patients and 66.1% had dyslipidemia. Patients in the analysis set (*n* = 32,492) differed from the total cohort of T2DM patients (*n* = 413,239) with a substantially higher use of metformin (56.7% vs. 35.7%) and sulfonylureas (19.4% vs. 10.6%), by a higher rate of hypertension (88.1% vs. 74.7%), and higher ACEi/ARB use (59.4% vs. 39.5%) (Table 1).

**Table 1:** Patient characteristics (*N* = 32,492)

	All patients with T2DM (*n* = 413,239) mean ± SD or %	Subgroup (*n* = 32,492) mean ± SD or %
Age, years	68.2 ± 12.9	66.3 ± 11.4
Male gender, %	52.8	52.6
Body mass index, kg/m^2^	31.0 ± 6.7	31.7 ± 6.2
Diabetes duration, years	10.5 ± 9.2	11.3 ± 8.5
HbA1c, %	7.6 ± 1.9	7.0 ± 1.0
Antidiabetic drug treatment		
Insulin, %	47.1	45.8
Metformin, %	35.7	56.7
DPP4-inhibitors, %	13.8	16.7
Sulfonylurea, %	10.6	19.4
Glinides, %	3.2	7.5
Acarbose, %	1.1	2.1
Sensitizers, %	1.0	4.5
GLP-1 analogues, %	2.9	6.6
SGLT-2 inhibitors, %	2.5	4.2
Hypertension, %	74.7	88.1
Syst. blood pressure, mmHg	135.4 ± 18.3	135.5 ± 12.5
Diast. blood pressure, mmHg	77.4 ± 10.6	78.6 ± 7.3
Antihypertensive drug treatment, %	52.1	67.0
ACEi, %	28.2	37.8
ARBs, %	11.3	21.6
Betablockers, %	28.4	35.1
Calcium channel blockers, %	14.7	23.7
Diuretics, %	28.7	31.8
Dyslipidemia, %	47.6	66.1
Kidney parameters		
eGFR, mL/min/1.73 m^2^	68.2 ± 26.5	76.7 ± 21.7
Serum potassium, mmol/L	4.3 ± 0.6	4.4 ± 0.5
Comorbidity at baseline		
Myocardial infarction, %	8.1	9.7
Stroke, %	7.3	8.0
Heart failure, %	5.7	9.2
Peripheral artery disease, %	16.3	32.8
Major amputation, %	0.9	0.8
Minor amputation, %	2.2	1.9
Diabetic neuropathy, %	43.6	69.5
Diabetic foot syndrome, %	11.6	25.1
Diabetic retinopathy, %	4.8	13.5

*Legend: DPP* dipeptidyl peptidase-4, *eGFR* estimated glomerular filtration rate, *GLP-1* glucagon-like peptide-1, *HDL* high-density lipoprotein, *LDL* low-density lipoprotein, *SGLT-2* sodium-glucose transport protein-2

### Renal function/eGFR slope

In terms of their renal function, 63.2% of the patients had macroalbuminuria, 34.0% had microalbuminuria and 2.8% macroalbuminuria (any albuminuria 36.8%). At a mean eGFR of 78.4 mL/min/1.73m^2^, 1.8% of all patients had an eGFR of < 30, 18.4% of between 30 and < 60, and 78.7% of ≥60 mL/min/1.73m^2^ (Table 2). Over a three-year time period, renal function deteriorated with an increase in the rate of albuminuria (+ 4.1%) and a decline of the eGFR with more than doubling of patients with an eGFR < 30 (3.8% vs. 1.8%; *p* < 0.001).

**Table 2:** Kidney parameters at baseline and throughout a 3-year follow-up (*N* = 32,492 at baseline)

	Baseline	at 1 year	at 2 years	at 3 years	Δ 3 years vs. baseline
Albuminuria					
Normoalbuminuria^a^, %	63.2	60.2	58.8	59.1	−4.1
Microalbuminuria, %	34.0	36.8	37.9	37.2	+ 3.2
Macroalbuminuria, %	2.8	3.0	3.3	3.7	+ 0.9
eGFR, ml/min/1.73m^2^	78.4 ± 21.4	77.6 ± 21.8	76.0 ± 22.2	74.5 ± 23.1	−3.9
eGFR ≥90, %	32.9	32.2	29.7	28.4	−4.5
eGFR 60 to < 90, %	45.8	46.4	46.4	45.2	−0.6
eGFR 45 to < 60, %	13.0	12.9	13.9	14.6	+ 1.6
eGFR 30 to < 45, %	5.4	6.3	7.1	8.0	+ 2.6
eGFR 15 to < 30, %	1.5	1.8	2.3	3.1	+ 1.6
eGFR < 15, %	0.3	0.5	0.5	0.7	+ 0.4
Chronic kidney disease^b^					
Low risk	33.0	32.2	29.7	28.4	−4.6
Moderate risk	46.8	46.4	46.4	45.2	−1.6
High risk	18.4	19.2	21.0	22.6	+ 4.2
Very high risk	1.8	2.2	2.9	3.8	+ 2.0

*Legend:*^a^Neither microalbuminuria nor macroalbumuria, but albuminuria below the microalbuminuria threshold possible. ^b^CKD was defined as eGFR < 60 mL/min/1.73 m^2^ OR eGFR ≥60 mL/min/1.73m^2^ and albuminuria (≥30 mg/g) [12, 13]. eGFR, estimated glomerular filtration rate

The eGFR slope, defined as a decrease of the eGFR over time, was highly variable (Table 3). Overall 54.3% patients had a decline of their eGFR within 3 years, with 30.9% having a decline of more than − 12 mL/min/1.73m^2^, and 23.4% a decline of between 0 and − 12 mL/min/1.73m^2^. Conversely, 45.7% of patients had a stable or increased eGFR.

**Table 3:** Patient characteristics (*N* = 32,492)

	eGFR-increase^a^ > 12 (27.1% of pts)	eGFR-increase^a^ 0 up to + 12 (18.6% of pts)	eGFR-slope^a^ 0 down to − 12 (23.4% of pts)	eGFR-slope^a^ − 12 or smaller (30.9% of pts)
Age, years	56.9 ± 10.2	66.9 ± 9.0	69.8 ± 8.5	74.1 ± 8.2
Male gender, %	57.3	57.5	51.2	43.9
Body mass index, kg/m^2^	32.5 ± 6.7	31.0 ± 5.8	31.2 ± 5.9	31.7 ± 6.0
Diabetes duration, years	9.1 ± 6.9	10.8 ± 8.0	11.7 ± 8.7	14.0 ± 9.5
HbA1c, %	7.0 ± 1.1	6.9 ± 0.9	6.9 ± 0.9	7.0 ± 0.9
Antidiabetic drug treatment				
Insulin, %	41.8	41.8	44.6	54.6
Metformin, %	68.6	61.8	58.4	37.5
DPP4-inhibitors, %	17.1	14.1	15.9	19.1
Sulfonylurea, %	18.1	20.6	20.3	19.3
Glinides, %	6.4	6.8	7.0	9.7
Acarbose, %	1.7	2.1	2.1	2.5
Sensitizers, %	4.8	4.6	4.2	4.3
GLP-1 analogues, %	11.2	5.8	4.8	3.3
SGLT-2 inhibitors, %	7.3	3.6	3.0	1.9
Hypertension, %	83.6	88.0	89.9	91.8
Syst. blood pressure, mmHg	134.9 ± 12.4	136.1 ± 12.3	135.8 ± 12.4	135.5 ± 12.7
Diast. blood pressure, mmHg	80.9 ± 7.1	78.9 ± 6.9	78.1 ± 7.0	76.2 ± 7.3
Antihypertensive drug treatment, %	60.0	66.5	69.5	73.9
ACEi, %	35.3	37.1	38.4	40.7
ARBs, %	18.0	20.6	22.1	26.2
Betablockers, %	26.4	32.4	37.8	45.5
Calcium channel blockers, %	18.5	22.5	24.1	30.5
Diuretics, %	19.6	26.9	32.7	49.5
Dyslipidemia, %	68.7	68.2	69.2	59.1
Kidney parameters				
eGFR, mL/min/1.73 m^2^	99.8 ± 8.6	83.5 ± 4.3	70.7 ± 4.5	48.5 ± 12.3
Serum potassium, mmol/L	4.4 ± 0.4	4.4 ± 0.4	4.4 ± 0.4	4.4 ± 0.5
Albuminuria				
Microalbuminuria, %	42.3	40.4	41.8	50.9
Macroalbuminuria, %	1.7	1.9	2.2	7.7
Comorbidity at baseline				
Myocardial infarction, %	5.4	8.9	10.3	14.8
Stroke, %	3.9	7.7	8.8	12.6
Heart failure, %	4.1	7.3	8.1	16.1
Peripheral artery disease, %	22.2	31.3	35.1	44.6
Major amputation, %	0.5	0.7	0.9	1.2
Minor amputation, %	0.9	1.4	1.9	3.4
Diabetic neuropathy, %	61.1	68.7	72.2	77.9
Diabetic foot syndrome, %	18.6	23.8	27.4	32.2
Diabetic retinopathy, %	9.6	11.6	14.3	19.1

*Legend:* values are mean ± SD; ^a^Within 3 years; DPP4, dipeptidyl peptidase-4; eGFR, estimated glomerular filtration rate; GLP-1, glucagon-like peptide-1; HDL, high-density lipoprotein; LDL, low-density lipoprotein; SGLT-2, sodium-glucose transport protein-2

Patients with a decline in renal function (slope of 12 or more; Table 3) had a compromised eGFR at baseline (48.5 mL/min/1.73m^2^), while patients with an increase in the eGFR were those with a normal renal function at baseline (mean eGFR 99.8 mL/min/1.73m^2^ in those with an increase of > 12; 83.5 mL/min/1.73m^2^ in those with an increase between 0 and 12). Furthermore, patients with a slope of more than − 12 were older (74.1 vs. 56.9 years), more often female (56.1 vs. 42.7%), with a longer diabetes duration (14.0 vs. 9.1 years), an increased rate of hypertension (91.8 vs. 83.6%), and had a higher rate of micro- (50.9 vs. 42.3%) and even more so macroalbuminuria (7.7 vs. 1.7%) than patients with an increased eGFR (> 12). This trend was consistent through all slope categories. Interestingly, rates of dyslipidemia were lower in those patients with a steep renal function decline.

### Albuminuria, hypertension and dyslipidemia

Albuminuria was indicative of a steeper slope of the eGFR. The prevalence of microalbuminuria was 50.9% in patients with a slope of more than − 12 mL/min/1.73m^2^ decline while it was in the order of 41% in patients with an eGFR change from − 12 to positive values (Table 4). This pattern persisted even after multivariable adjustment for age, sex, diabetes duration, BMI and ACEi / ARB use, with a *p*-value < 0.001 for the difference across eGFR slope categories.

**Table 4:** Association between albuminuria, hypertension, dyslipidemia and GFL-slope categories

	Univariate frequency (95%CI)	Model 1 frequency (95%CI)	Model 2 frequency (95%CI)	***p***-value Model 2
Microalbuminuria (%)				
eGFR-increase^a^ > 12	42.3 (41.3–43.4)	42.9 (41.7–44.1)	42.8 (41.6–44.0)	< 0.001
eGFR-increase^a^ 0 up to + 12	40.4 (39.2–41.6)	40.3 (39.1–41.5)	40.3 (39.1–41.5)	
eGFR-slope^a^ 0 down to −12	41.8 (40.5–43.2)	41.4 (40.1–42.8)	41.3 (40.0–42.7)	
eGFR-slope^a^ -12 or smaller	50.9 (49.7–52.0)	49.4 (48.2–50.7)	49.1 (47.9–50.4)	
Hypertension (%)				
eGFR-increase^a^ > 12	83.6 (82.9–84.4)	88.4 (87.7–89.1)	n.a.	0.002^b^
eGFR-increase^a^ 0 up to + 12	88.0 (87.3–88.8)	89.0 (88.2–89.7)	n.a.	
eGFR-slope^a^ 0 down to −12	89.9 (89.2–90.7)	89.9 (89.0–90.6)	n.a.	
eGFR-slope^a^ < −12 or smaller	91.8 (91.3–92.4)	90.4 (89.7–91.1)	n.a.	
Dyslipidemia (%)				
eGFR-increase^a^ > 12	68.7 (67.7–69.6)	70.7 (69.6–71.7)	72.4 (71.3–73.4)	< 0.001
eGFR-increase^a^ 0 up to + 12	68.2 (67.1–69.2)	68.2 (67.1–69.3)	69.9 (68.8–71.0)	
eGFR-slope^a^ 0 down to −12	69.2 (68.0–70.4)	69.0 (67.8–70.2)	70.4 (69.2–71.6)	
eGFR-slope^a^ < −12 or smaller	59.1 (58.1–60.2)	58.9 (57.7–60.0)	58.7 (57.5–60.0)	

*Legend:*^a^Within a time span of 3 years; ^b^p-value Model 1; Model 1 adjusted for age, sex, duration and BMI; Model 2 adjusted for model 1 variables plus ACE-inhibitor use and/or ARB use

The presence of hypertension also adds risk to a decline of renal function. At an overall hypertension prevalence of 88.1%, hypertension rates were 91.8% in those with an eGFR slope of > 12. This pattern is slightly alleviated, but still retained if numbers are adjusted for age, sex, duration and BMI with a p-value of 0.002. ACEi/ARB use adjustment was not performed as the use of antihypertensive drugs interferes with the definition of hypertension.

Dyslipidemia, defined as an LDL-C of at least 100 mg/dL or at least 70 mg/dL in the presence of CVD/CKD, was noted in 66.1% of patients. Rates were lower in patients with a steep eGFR slope (− 12 or smaller) and higher in those with a retained eGFR. This pattern persisted after adjustment for age, sex, diabetes duration, BMI and ACEi / ARB use (*p* < 0.001).

In a composite analysis the presence of microalbuminuria (− 1.51; 95% CI − 1.81 to 1.22) and the absence of dyslipidemia (− 3.82; 95% CI − 4.20 to 3.45) had the highest impact on the progression of an eGFR decline. This was up and beyond the effects of age, gender, diabetes duration and BMI.

## Discussion

The results of the present analysis show that the prevalence of renal function impairment understood as CKD is considerable and has a high incidence rate within a year. Serial determinations of the glomerular filtration rate are, however, infrequent and these patients are characterised by the presence of a high-risk profile for CKD, such as hypertension and dyslipidemia. Moreover, the use of renin-angiotensin blocking agents were low, pointed at considerable room for improvement. Over a three-year time period, one-third of these patients had an eGFR slope of − 12 or more and a more than doubled proportion of patients with an eGFR < 30 mL/min/1.73 m^2^. Hypertension and albuminuria contributed to renal function decline while dyslipidemia did not negatively affect the slope.

### Prevalence and incidence of CKD

We based our definition of CKD on the decline of renal function (eGFR < 60 mL/min/1.73m^2^) and the presence of urinary albumin. As previously published, our cohort of T2DM patients is characterized by a CKD prevalence in the order of 50.0% [2], based on data obtained up until March 2018. While the rate of 53.0% in the current sample as of March 2019 may be numerically higher, it is not very likely that it represents a more general trend. The order, however, is consistent with other publications when different definitions and patient populations are considered. Gonzalez-Perez et al. reported that 29,104 out of 109,365 patients with newly diagnosed T2DM (26.6%) already had CKD with an incidence rate of 5.5 per 100 years [15]. Zelnick et al. reported, for patients with diabetes, a prevalence of 25% [16]. While the former reported rates in patients with newly diagnosed diabetes, the latter excluded those with albuminuria at a GFR of > 60 mL/min/1.73m^2^. As such, we believe that a prevalence rate in the order of 50% is a good estimate of the true prevalence in T2DM, which is reconfirmed by others [17].

### Renal function decline

We would assume that, in patients with T2DM, there is a linear decline in renal function over time eventually leading to end-stage renal disease and dialysis in a subset of patients. The GFR decline in T2DM patients is almost twice as high as in patients without diabetes [18]. This is even more true with an ageing population as patients increasingly survive cardiovascular events and have sufficient time to develop renal disease [17]. The linearity of this decline has been challenged more recently with data showing a non-linear or even non-progression in patients with CKD [19]. While this research was performed in patients without diabetes, Weldegiorgis et al. suggested than non-linearity may be particularly frequent in those with diabetes [20]. These data are in full alignment with our own findings. The proportion of patients with an eGFR < 30 mL/min/1.73 m^2^ doubled within the observational period of 3 years in the subset of patients where the treating physician performed a closer surveillance of the renal function (3.8% vs. 1.8%; *p* < 0.001). Overall, 30.9% of the patients had an eGFR slope of − 12 or more which is consistent with the general renal function decline, but, on the other hand, a substantial proportion of patients also had a more or less stable decline or even eGFR increase which was mostly observed in those patients with a normal or just mildly impaired eGFR at baseline. This is potentially in agreement with the initial hyperfiltration observed in early renal function decline. While this may be influenced by the variability of eGFR determinations itself [21], it should also be regarded as a reflection of the non-linear decline of renal function in patients with diabetes [22].

### Renal function decline cofactors

There is a considerable overlap between patients with reduced eGFR and those showing varying degrees of urinary albumin excretion. While there are patients in stage 3 CKD but with normoalbuminuria [23–25] (26.2% in our population), there are also patients without or with only mild reductions in the eGFR, but showing varying levels of urinary albumin excretion (14.6% in our cohort). The presence of albuminuria in patients with reduced GFR has been associated with progressive kidney disease [26] and confers additional risk [2, 12]. Consistent with this research, renal function decline in our dataset was pronounced in the presence of albuminuria with a faster deterioration in patients with macroalbuminuria than microalbuminuria, which is in line with recent meta-analyses [26, 27].

Hypertension prevalence was higher in patients with accelerated renal function decline in our dataset. It is a well-recognized risk factor and antihypertensive treatment, especially the use of ACEi or ARBs is considered to slow the eGFR decline. This was recently reconfirmed in an analysis of the *Atherosclerosis Risk in Communities* (ARIC) study [28]. Compared to normotension, hypertension status was associated with faster kidney function decline over 30-year follow-up in a general population cohort. This difference was attenuated among people using antihypertensive medications.

We were surprised by a lack of an association or even a reverse relationship between dyslipidemia and eGFR slope. We defined dyslipidemia as an LDL-C cholesterol of ≥100 mg/dL without further risk factors and ≥ 70 mg/dL in patients with CVD or CKD or patients receiving lipid-lowering drug treatment. The definition resulted in higher dyslipidemia rates in those with CKD, but there was no increase in the eGFR slope seen. The results are difficult to interpret, as research on the relationship between dyslipidemia and GFR function decline is sparse. One of the few studies is a retrospective Japanese study in 4326 patients that shows an association of dyslipidemia with the deterioration of proteinuria and renal function. The authors found that neither total cholesterol nor LDL-C (but high triglyceride levels) were associated with renal function decline [29]. Moreover, it appears that lipid-lowering treatment is usually considered not to be associated with renal function decline, but is mandatory to ameliorate the adverse long-term cardiovascular outcomes [30]. Actually, in our own dataset we found comparable levels of total cholesterol and LDL-C, while triglycerides were higher in those with renal function decline.

### Guideline considerations

Early detection and treatment of CKD may delay or prevent the development of end-stage kidney disease, morbidity, and mortality. Aiming at the improvement of early CKD detection, the Kidney Disease Outcomes Quality Initiative (K/DOQI) of the National Kidney Foundation published clinical practice guidelines recommending the use of estimating equations of GFR on the basis of serum creatinine determinations and Urinary-Albumin-Creatinine-Ratio (UACR) [12, 13]. The American Diabetes Association (ADA, 2020) [31] recommends spot urinary albumin-to-creatinine ratio, serum creatinine and estimated glomerular filtration rates evaluations at baseline and then annually. This approach was recently reinforced by the ESC in collaboration with the EASD (2020) [32]. Although there is no formal guidance from the American Society of Nephrology (ASN) they strongly advocate “regular screening for kidney disease regardless of risk factors” [33, 34], similar to a statement published by the National Kidney Foundation (NKF) and the Renal Physicians Association (RPA) [35].

Based on our own observations, more than 90% of patients with diabetes in Germany received no regular kidney disease screening. Patients that were screened were at increased risk, based on their patient profile as diabetic patients and had a higher prevalence of concomitant hypertension and microalbuminuria. While the adequacy of this approach deserves further investigation it appears that targeting at risk patients is a viable strategy to detect patients with kidney disease early. Australia, Canada, Japan, UK, and the USA have established such effective surveillance mechanisms for chronic kidney disease in an attempt to detect the disease early and improve outcomes [36–38]. A recent research project investigated the effects of a virtual CKD clinic (VC) in patients with CKD. It consisted of a non-face-to-face computer-assisted review of patient data and was associated with improved survival compared to standard care and a reduction in patients requiring emergency dialysis [39].

### Limitations

The current registry analysis reflects real-world diagnostic and treatment patterns in a very large group of patients with T2DM, which is representative for patients treated in Germany. As such, it gives valuable insight up and beyond clinical trials into patient groups that were potentially never studied in clinical trials. We found that only 7.8% of the patients received serial eGFR determinations over a time frame of 3 years, which reflects clinical practice, but also defines a subset of patients where physicians felt particular attention was needed. It appears as if antiproteinuric, as well as antihypertensive, treatment provides benefit, while lipid-lowering drugs may not ameliorate the progression of kidney disease.

## Conclusions

Chronic kidney disease is highly prevalent in a T2DM patient population. Serial surveillance of the glomerular filtration rate is, however, not routinely established in clinical practice, which would be necessary as indicated by a doubling of patients with an eGFR < 30 mL/min/1.73 m^2^ within 3 years. Moreover, the use of renin-angiotensin blocking agents was low, pointing at considerable room for improvement. Taken together we conclude that a closer surveillance of patients with diabetes based on the presence of further risk factors is mandatory combined with a mandatory prescription of RAS blocking agents once microalbuminuria and / or renal function deterioration develops.

## Data Availability

The datasets generated and analyzed during the current study are not publicly available due to data privacy but are available from the corresponding author on reasonable request.

## Abbreviations

ACEi: ACE-inhibitor
ARB: Angiotensin receptor blocker
BMI: Body mass index
BP: Blood pressure
CI: Confidence Interval
CKD: Chronic kidney disease
CKD-EPI: Chronic Kidney Disease Epidemiology Collaboration
CVD: Cardiovascular disease
DBP: Diastolic blood pressure
eGFR: Estimated glomerular filtration rate
ESRD: End-stage renal disease
GFR: Glomerular filtration rate
LDL-C: Low density lipoprotein cholesterol
SBP: Systolic blood pressure
T2DM: Type 2 diabetes mellitus
